# Mortality and potential years of life lost attributable to alcohol consumption in Canada in 2005

**DOI:** 10.1186/1471-2458-12-91

**Published:** 2012-01-31

**Authors:** Kevin D Shield, Benjamin Taylor, Tara Kehoe, Jayadeep Patra, Jürgen Rehm

**Affiliations:** 1 Centre for Addiction and Mental Health (CAMH), Toronto, Canada; 2 Dalla Lana School of Public Health (DLSPH), University of Toronto, Toronto, Canada; 3 Department of Statistics, University of Toronto, Toronto, Canada; 4 Institute for Clinical Psychology and Psychotherapy, TU Dresden, Germany

**Keywords:** Alcohol consumption, Mortality, Potential years of life lost, Relative risk, Canada

## Abstract

**Background:**

Alcohol is a substantial risk factor for mortality according to the recent 2010 World Health Assembly strategy to reduce the harmful use of alcohol which outlined the need to characterize and monitor this burden. Accordingly, using new methodology we estimated 1) the number of deaths caused and prevented by alcohol consumption, and 2) the potential years of life lost (PYLLs) attributable to alcohol consumption in Canada in 2005.

**Methods:**

Mortality attributable to alcohol consumption was estimated by calculating Alcohol-Attributable Fractions (AAFs) (defined as the proportion of mortality that would be eliminated if the exposure was eliminated) using data from various sources. Indicators for alcohol consumption were obtained from the Canadian Alcohol and Drug Use Monitoring Survey 2008 and corrected for adult *per capita *recorded and unrecorded alcohol consumption. Risk relations were taken from the Comparative Risk Assessment within the current Global Burden of Disease (GBD) study. Due to concerns about the reliability of information specifying causes of death for people aged 65 or older, our analysis was limited to individuals aged 0 to 64 years. Calculation of the 95% confidence intervals (CIs) for the AAFs was performed using Monte Carlo random sampling. Information on mortality was obtained from Statistics Canada. A sensitivity analysis was performed comparing the mortality results obtained using our study methods to results obtained using previous methodologies.

**Results:**

In 2005, 3,970 (95% CI: 810 to 7,170) deaths (4,390 caused and 420 prevented) and 134,555 (95% CI: 36,690 to 236,376) PYLLs were attributable to alcohol consumption for individuals aged 0 to 64 years. These figures represent 7.7% (95% CI: 1.6% to 13.9%) of all deaths and 8.0% (95% CI: 2.2% to 14.1%) of all PYLLs for individuals aged 0 to 64 years. The sensitivity analysis showed that the number of deaths as measured by this new methodology is greater than that if mortality was estimated using previous methodologies.

**Conclusions:**

The mortality burden attributable to alcohol consumption for Canada is large, unnecessary, and could be substantially reduced in a short period of time if effective public health policies were implemented. A monitoring system on alcohol consumption is imperative and would greatly assist in planning and evaluating future Canadian public health policies related to alcohol consumption.

## Background

Alcohol consumption is responsible for substantial morbidity, mortality, and social problems in both developing and developed countries [1,2]. Alcohol has been associated with more than 230 International Classification of Diseases version 10 (ICD 10) codes [2-5] and is estimated to be the third most common cause of disability adjusted life years lost (DALYs), responsible for 4.5% of the DALYs worldwide [1]. In light of the considerable harm associated with alcohol consumption as reflected in these figures, member nations of the World Health Organization (WHO) agreed during the 63^rd ^World Health Assembly held in May 2010 to a global strategy for reducing the harmful use of alcohol [6]. The recommendations outlined in the global strategy include that an appropriate level of attention be accorded to alcohol consumption, that the importance of strengthening information about alcohol consumption and alcohol-related harms be recognized, and that this information be effectively disseminated. Each member nation of the WHO has the responsibility to monitor its country's alcohol consumption and alcohol-related harms data (see also [7]).

Mortality caused by alcohol consumption in a population can be estimated using an Alcohol-Attributable Fraction (AAF). The AAF is defined as the proportion of mortality that would be prevented if the exposure to alcohol was completely eliminated from the population, and can be expressed as follows:

(1) $$AAF=\frac{\overset{k}{\underset{i=0}{\sum }}P_{i}\left(RR_{i}-1\right)}{\overset{k}{\underset{i=0}{\sum }}P_{i}\left(RR_{i}\right)}$$

where P_i _represents the proportion of people in exposure group i, and RR_i _is the relative risk of mortality for group i compared to the reference group (in alcohol research, often non-drinkers or lifetime abstainers). The AAF is typically calculated for a number of drinking categories (from i = 0 to k, where i = 0 is the reference group and k + 1 is the number of drinking categories). Previous papers that estimated the number of deaths attributable to alcohol used categorical estimates of alcohol consumption by calculating the AAFs for various diseases [8,9]. Alcohol consumption displays a right-skewed distribution; calculating the number of deaths caused and prevented by alcohol using categorical or log transformation techniques will not completely adjust for this right-skew, resulting in an underestimation of mortality and morbidity [8,10,11]. In this paper, mortality estimates for Canada in 2005 are presented using new risk modelling methods that take into account the right-skewed distribution of alcohol consumption obtained from survey data. This method was developed as part of the ongoing Global Burden of Disease (GBD) Comparative Risk Assessment and the US Burden of Disease Study (see Murray et al.[12] for GBD; see Rehm et al. [3,10] for a description of methodology).

In accordance with the World Health Assembly global strategy, the objectives of this paper are 1) to estimate the number of deaths caused and prevented by alcohol consumption in Canada in 2005, and 2) to calculate the potential years of life lost (PYLLs) attributable to alcohol in Canada in 2005. Further objectives of this study are 3) to establish trends in alcohol-attributable mortality, and 4) to compare the methods used in this paper with previous methods used to calculate alcohol-attributable mortality in Canada [8,13].

## Methods

In order to estimate alcohol-attributable harms as outlined in the objectives, we collected data on the measurement of the exposure, the risk relationships (RRs) and AAFs, and the measurement of the outcome, namely, mortality.

### Exposure estimates

Alcohol consumption estimates were determined using data derived from the Canadian Alcohol and Drug Use Monitoring Survey (CADUMS) 2008 [14]. The CADUMS 2008 was used since it is the largest nationally representative survey on alcohol consumption and was completed most recent to, but after, 2005. Furthermore, future iterations of this survey are planned, so comparisons of exactly the same questions over time should be possible. The survey methods utilized for the CADUMS 2008 are described in detail elsewhere [15], but, briefly, the CADUMS 2008 was a random digit dialing telephone-based survey conducted over 8 months. In total, 43,328 individuals were contacted, of whom 16,674 responded. Of those who responded, 16,640 individuals provided a valid age and sex. *A posteriori *weighting of those individuals who responded was performed by sex, age, and region by triangulating the survey information with the 2006 Canadian census. Of the participants who were weighted, 15,801 provided a valid alcohol response and age category, resulting in an overall participation rate of 36.5%. When compared to the adult *per capita *consumption of recorded alcohol in Canada in 2008, the CADUMS 2008 coverage rate was 34%.

Drinking status in the CADUMS 2008 was defined as "current drinkers" (individuals who consumed at least one drink in the past year), "former drinkers" (individuals who consumed at least one drink in their lifetime, but not within the last year), and "lifetime abstainers" (individuals who had never consumed a drink in their lives). Alcohol intake for "current drinkers" was measured in the CADUMS 2008 in terms of standard drinks over the 7 days prior to the survey. A standard drink for Canada was defined as 13.6 g of alcohol [16]. Since the CADUMS 2008 did not measure alcohol consumption in individuals younger than 15 years of age, and the amount of alcohol consumed by people in this age category is considered to be negligible, in our analysis we categorized everyone younger than 15 years of age as a lifetime abstainer. For binge drinking amounts we used estimates from the 2001 and 2002 waves of the National Epidemiological Survey on Alcohol and Related Conditions (NESARC) as an estimate for Canadians [17].

The prevalence of alcohol consumption during pregnancy was estimated to be 13.3%, as obtained from the Public Health Agency of Canada [18]. Average daily consumption of alcohol by women who are pregnant was calculated as a weighted average (weighted in proportion to prevalence of births among women of different age groups).

The estimated adult *per capita *consumption of recorded and unrecorded alcohol consumption for Canada in 2005 was based on information regarding taxation and alcohol production export and import data, generally considered to be the best estimate of overall alcohol consumption in high-income countries [19]. *Per capita *consumption estimates for unrecorded consumption were calculated to be 19.5% of total alcohol consumption, based on the research of Macdonald and colleagues [20].

### Upshifting and modelling alcohol consumption

To account for the undercoverage seen in the CADUMS 2008, we upshifted the mean intake in grams per day by triangulating the data with *per capita *consumption estimates of recorded and unrecorded alcohol for Canada in 2005. This was achieved by multiplying the unshifted mean (for each sex and age group) by the inverse of 90% of the coverage rate. As recommended by Rehm and colleagues [10], we chose the inverse of 90% of the coverage rate in order to account for alcohol not consumed due to, for example, spillage, waste and breakage, and to account for undercoverage of reported alcohol consumption that was most likely present in the observational studies used in the meta-analyses from which we obtained our RR estimates.

By using 1,001 alcohol distributions from 66 different countries, Rehm and colleagues have shown that alcohol consumption data from population surveys are best continuously modelled using a Gamma distribution [10]. We thus used a Gamma distribution to model upshifted alcohol consumption.

To model upshifted alcohol consumption in our study, we also used the relationship between the mean alcohol consumption and standard deviation of the alcohol distribution outlined by Rehm and colleagues (calculated by regressing over 500 means (μ) and standard deviations (σ)). This relationship can be expressed as follows:

(2) $$\sigma_{men}=1.171*\mu_{men}$$

(3) $$\sigma_{women}=1.258*\mu_{women}$$

Using these relationships, we calculated the standard deviation of the upshifted Gamma distributions and then used the mean and standard deviation to calculated shape and scale parameters which are used to describe the upshifted Gamma distribution [10].

### Relative risks

The sources for the RRs are provided in the Additional file 1. An outline of the causal relationship between alcohol consumption and all alcohol-related causes of morbidity and mortality is described by Rehm and colleagues [3]. Alcohol-attributable deaths were calculated only for chronic diseases and injuries where a meta-analysis reported the existence of a continuous RR function.

### Mortality data

Data on the number of deaths in Canada in 2005, coded by the ICD 10, were obtained from Statistics Canada; 2005 data were the most current available [21]. Table 1 lists the causes of death where alcohol plays a causal role and where a meta-analysis exists quantifying the continuous risk of the disease given a level of alcohol consumption. Deaths in individuals over the age of 64 were not used in our main analysis, since specified causes of death after this age were not considered reliable; however, when comparing the estimated deaths with previous methods (see below in "*Comparisons with previous studies*"), age categories above 64 were included in mortality and PYLLs estimates.

**Table 1:** Prevalence of alcohol consumption in Canada 2008 according to age, gender

		Abstainers		Former drinkers		Current drinkers	
**Gender**	**Age**	**Percent**	**95% CI**	**Percent**	**95% CI**	**Percent**	**95% CI**

Women	15-24	19.0	(13.4-24.5)	7.0	(3.2-10.7)	74.1	(67.9-80.3)

	25-34	10.4	(6.8-14.0)	11.9	(8.5-15.2)	77.8	(73.2-83.3)

	35-44	8.0	(5.4-10.5)	12.5	(9.5-15.5)	79.6	(75.8-83.3)

	45-54	8.0	(5.5-10.4)	14.9	(11.9-17.9)	77.2	(73.5-80.8)

	55-64	9.7	(7.2-12.2)	16.3	(13.3-19.3)	74.0	(70.4-77.6)

	65-74	12.9	(9.5-16.3)	19.0	(14.8-23.3)	68.0	(63.0-73.0)

	75+	27.2	(21.8-32.5)	21.7	(16.8-26.5)	51.2	(45.2-57.1)

Men	15-24	13.1	(8.9-17.3)	3.4	(1.2-5.6)	83.5	(78.9-88.1)

	25-34	5.8	(2.3-9.3)	9.4	(4.7-14.0)	84.9	(79.3-90.4)

	35-44	5.3	(2.9-7.6)	8.6	(5.8-11.3)	86.2	(82.7-89.7)

	45-54	5.3	(3.1-7.5)	12.0	(8.8-15.1)	82.8	(79.1-86.4)

	55-64	3.7	(2.0-5.4)	17.7	(13.6-21.9)	78.5	(74.2-82.9)

	65-74	8.0	(4.2-11.8)	22.8	(16.7-28.9)	69.2	(62.5-75.8)

	75+	7.5	(3.2-11.8)	20.0	(12.6-27.4)	72.5	(64.4-80.6)

Total		9.8	(8.9-10.7)	12.9	(11.9-13.8)	77.3	(76.1-78.6)

### Potential Years of Life Lost

PYLLs were calculated for Canada using the age specific number of deaths in Canada prevented and caused by alcohol consumption as determined by subtracting the average age of death from the average life expectancy for the age categorization, and by then multiplying this number by the number of alcohol-attributable deaths within the given age group. Age groups were cut off at age 64 since specified causes of death after this age were not considered reliable. The GBD study guidelines were followed when calculating PYLLs values [22].

### Calculating AAFs

The AAF for each cause of death and morbidity was calculated by sex and age, taking into account the distribution of alcohol consumption and the prevalence of different drinking statuses ("current drinkers," "former drinkers," and "lifetime abstainers") as follows:

(4) $$AAF=\frac{P_{abs}+P_{form}RR_{form}+\overset{150}{\underset{0}{\int }}P\left(x\right)RR\left(x\right)dx-1}{P_{abs}+P_{form}RR_{form}+\overset{150}{\underset{0}{\int }}P\left(x\right)RR\left(x\right)dx}$$

where P_abs _represents the proportion of "lifetime abstainers," P_form _the proportion of "former drinkers," and P(x) the probability distribution function of drinkers. RR_form _represents the relative risk (RR) for "former drinkers," and RR(x) the RR function for a given alcohol consumption in grams per day. A cap at an exposure of 150 g of pure alcohol was used as a conservative measure, as very few individuals consume more than 12 standard drinks on a daily basis for an extended period of time; those individuals were modelled as drinking 150 g.

The RRs for injuries were calculated according to Taylor and colleagues by taking into account average consumption, occasions of binge drinking, and the length of time at risk after consumption (based on metabolism data) [23]. The AAFs for average consumption were calculated using formula 4, which incorporated an adjusted RR for the time at risk; this adjusted RR value is calculated as follows:

(5) $$RR\left(x\right)=P_{dayatrisk}*P_{daysatrisk}*\left(RR_{Crude}\left(x\right)-1\right)+1$$

where P_dayatrisk _(calculated based on alcohol intake × in grams per day) represents the proportion of a given day during which a person drinks and is at risk, P_daysatrisk _(for average consumption this was set at 1) represents the percentage of days the person undertakes drinking (binge or average), and RR_crude _represents the unadjusted risk ratio for injury given an alcohol consumption level of x.

For binge consumption the AAFs for injuries were calculated as follows:

(6) $$AAF=\frac{P_{abs+former}+P_{current\left(Non-Binge\right)}+P_{current\left(Binge\right)}RR\left(x\right)-1}{P_{abs+former}+P_{current\left(Non-Binge\right)}+P_{current\left(Binge\right)}RR\left(x\right)}$$

where P_abs+former _is the proportion of lifetime abstainers and former drinkers, and P_current(Binge) _and P_current(Non-Binge) _are the prevalences of current drinkers who engage and who do not engage in binge drinking, respectively. RR_binge_(x) represents the risk ratio for binge drinkers given a binge amount of alcohol consumed, corrected for both time at risk and number of drinking occasions.

### Calculating the 95% confidence intervals for the AAFs

95% CIs for the AAFs were calculated from the variance of 10,000 simulations [24]. For each simulation, estimates were generated for the prevalence of "current drinkers" and "former drinkers." For all conditions except injuries, a population mean was then calculated based on the generated prevalence of "current drinkers" and the generated mean alcohol consumption per day. This population mean was then upshifted by triangulating the generated mean alcohol consumption with the adult *per capita *consumption estimate. The standard deviation (SD) of the upshifted Gamma distribution, as well as the shape parameter estimates, were calculated based on the relationship between the upshifted mean and the SD as reported by Rehm and colleagues [10]. For instances of low birth weight, the prevalence of drinkers during pregnancy was assumed to be 13.3% as reported by the Public Health Agency of Canada; we assumed that mothers who consumed alcohol during pregnancy displayed a similar distribution to the upshifted distribution of drinkers [18]. The 10,000 AAFs for injuries were calculated based on simulations which generated estimates for average consumption, prevalence of current drinkers, binge consumption, prevalence of binge drinkers, and number of drinking occasions for people who engage in binge drinking.

### Comparisons with previous studies

The gross number of deaths and the PYLLs for Canada for 2005 (excluding the number of deaths and PYLLs prevented by alcohol) were estimated according to methods described by Rehm and colleagues [8,10]. For comparability with previous studies, the same age categorizations were used [8,13]. 15,531 participants of the CADUMS 2008 provided a valid, exact age, and information concerning their alcohol consumption, and were therefore included in our analysis. Gross estimates of attributable mortality were calculated which did not include data regarding mortality or PYLLs prevented by alcohol.

All statistics were performed using R version 2.10.1.

## Results

Tables 1 and 2 provide an overview of 1) the estimated volume of alcohol consumption, both unshifted estimates and upshifted estimates, 2) the prevalence of "current drinkers," "former drinkers," and "lifetime abstainers," and 3) the average number of drinking occasions per week by age and sex. As expected, men drank more than women, and alcohol consumption was the highest in the younger age groups.

**Table 2:** Consumption by age before and after upshifting estimates

		Raw estimates (Current Drinkers)		Corrected estimates (Current Drinkers		Number of drinking occasions per week	
**Gender**	**Age**	**Mean (g/day)**	**95% CI**	**Mean (g/day)**	**95% CI**	**Number**	**95% CI**

Women	15-24	9.67	(2.1-17.2)	26.4	(24.4-28.3)	0.80	(0.58-1.02)

	25-34	5.46	(3.5-7.4)	18.2	(16.8-19.5)	0.75	(0.61-0.88)

	35-44	3.63	(3.1-4.2)	12.1	(11.6-12.7)	0.82	(0.69-0.95)

	45-54	5.03	(4.1-5.9)	16.7	(16.0-17.4)	1.03	(0.89-1.18)

	55-64	4.55	(3.9-5.2)	15.1	(14.4-15.9)	1.06	(0.91-1.12)

	65-74	4.57	(3.4-5.7)	15.2	(14.0-16.4)	1.01	(0.77-1.26)

	75+	3.97	(2.9-5.0)	13.2	(12.0-14.5)	0.83	(0.59-1.07)

Men	15-24	11.68	(8.3-15.0)	38.5	(35.5-41.6)	0.92	(0.73-1.12)

	25-34	12.80	(9.8-15.8)	40.0	(36.6-43.3)	1.39	(1.14-1.64)

	35-44	9.14	(7.5-10.8)	29.7	(28.1-31.3)	1.40	(1.21-1.59)

	45-54	12.01	(8.8-15.2)	36.4	(34.4-38.4)	1.63	(1.42-1.86)

	55-64	10.58	(8.7-12.4)	34.7	(32.6-36.7)	1.68	(1.42-1.93)

	65-74	11.34	(7.5-15.2)	37.5	(34.1-40.9)	1.52	(1.15-1.89)

	75+	7.92	(5.7-10.1)	25.9	(23.1-28.7)	1.77	(1.29-2.25)

Total		5.79	(4.6-7.0)	-	-	1.15	(1.09-1.21)

Table 3 provides the net number of alcohol-attributable deaths caused (represented by a positive number) or prevented (represented by a negative number) by alcohol consumption in Canada in 2005. The 3,958 net alcohol-attributable deaths among individuals between the ages of 0 and 64 constituted 7.7% (95% CI: 1 6% to 13.9%) of all deaths in Canada for these age groups in 2005. This can be broken down into 9 net deaths for Canadians between the ages of 0 and 14 (8 deaths attributable to low birth weight due to alcohol consumption by the mother during pregnancy, and one death due to ethanol and methanol toxicity, undetermined intent) and 3,949 deaths for people between the ages of 15 and 64. Overall, there were 4,382 (95% CI: 2,967 to 7,184) deaths attributable to alcohol (1,111 (25.4%) for women; 3,271 (74.6%) for men), and 424 (95% CI: 0 to 2,154) deaths prevented by alcohol (108 (25.3%) for women; 316 (74.7%) for men). The largest contributors to alcohol-attributable mortality were unintentional injuries, malignant neoplasms, and digestive diseases. The most important specified causes of preventable deaths attributed to alcohol consumption were liver cirrhosis (868 deaths; 595 males, 273 females) and motor vehicle accidents (663 deaths; 588 males, 75 females).

**Table 3:** Alcohol-attributable mortality for Canada in 2005

	0 - 14 years		15 - 24 years		25 - 34 years			35 - 44 years		45 - 54 years		55 - 64 years		Total	
**Condition**	**M**	**F**	**M**	**F**	**M**	**F**	**M**	**F**	**M**	**F**	**M**	**F**	**M**	**F**	**Overall**

Total, all causes of death															

Total, all causes of death	5	4	375	84	355	46	429	102	843	345	948	422	2955	1003	3958

Malignant neoplasm's															

Mouth and oropharynx cancers	0	0	1	0	2	1	11	4	53	13	93	14	160	32	192

Esophageal cancer	0	0	0	0	1	0	6	0	38	5	102	10	147	15	162

Liver cancer	0	0	0	0	2	0	4	0	23	6	38	11	67	17	84

Laryngeal cancer	0	0	0	0	0	0	1	0	14	2	31	3	46	5	51

Breast cancer	0	0	0	0	0	5	0	35	0	114	0	148	0	302	302

Colon cancer	0	0	0	0	1	0	3	2	15	11	41	27	60	40	100

Rectum cancer	0	0	0	0	0	0	2	1	7	4	16	6	25	11	36

Total	0	0	1	0	6	6	27	42	150	155	321	219	505	422	927

Diabetes															

Total (diabetes mellitus)	0	0	0	3	0	1	-2	-4	1	15	7	12	6	27	33

Neuro-psychiatric conditions															

Alcoholic psychoses	0	0	2	1	5	1	13	2	19	8	27	8	66	20	86

Alcohol abuse	0	0	1	0	3	1	11	6	30	10	30	14	75	31	106

Alcohol dependence syndrome	0	0	0	0	2	1	22	6	74	19	65	20	163	46	209

Degeneration of nervous system due to alcohol	0	0	0	0	0	0	0	0	2	0	3	0	5	0	5

Epilepsy	0	0	4	2	7	2	7	2	9	5	6	3	33	14	47

Alcohol polyneuropathy	0	0	0	0	0	0	0	0	0	0	0	0	0	0	0

Total	0	0	7	3	17	5	53	16	134	42	131	45	342	111	453

Cardiovascular disease															

Hypertensive disease	0	0	0	0	0	0	3	0	15	3	16	6	34	9	44

Ischemic heart disease	0	0	0	0	-5	-1	-37	-5	-104	-16	-168	-33	-314	-55	-369

Alcohol cardiomyopathy	0	0	0	0	0	0	4	0	13	1	16	5	33	6	39

Cardiac arrhythmias	0	0	0	1	1	0	2	1	7	3	16	5	26	10	37

Ischemic stroke	0	0	0	0	1	-1	1	-9	6	-16	12	-23	20	-49	-29

Hemorrhagic and other non-ischemic stroke	0	0	0	0	0	1	3	2	11	9	47	19	61	31	93

Total	0	0	0	1	-3	-1	-24	-11	-52	-16	-61	-21	-140	-48	-185

Digestive diseases															

Alcoholic gastritis	0	0	0	0	0	0	1	0	2	0	2	0	5	0	5

Cirrhosis of the liver	0	0	0	0	8	5	69	33	217	105	302	130	596	273	868

Acute and chronic pancreatitis	0	0	0	1	0	0	1	0	9	1	10	2	20	4	25

Chronic pancreatitis (alcohol-induced)	0	0	0	0	1	0	3	1	5	2	6	1	15	4	19

Total	0	0	0	1	9	5	74	34	233	108	320	133	636	281	917

Conditions arising during the prenatal period															

Low birth weight: as defined by the global burden of disease	4	4	0	0	0	0	0	0	0	0	0	0	4	4	8

Fetal alcohol syndrome (dysmorphic)	0	0	0	0	0	0	0	0	0	0	0	0	0	0	0

Excess alcohol blood levels	0	0	0	0	0	0	0	0	0	0	0	0	0	0	0

Total	4	4	0	0	0	0	0	0	0	0	0	0	4	4	8

Unintentional injuries															

Motor vehicle accidents	0	0	205	46	137	12	78	3	105	8	62	6	587	75	664

Poisonings	0	0	10	3	27	3	35	3	34	5	8	2	114	16	130

Falls	0	0	6	0	5	0	6	0	17	1	21	2	55	3	58

Fires	0	0	1	1	2	0	2	0	5	1	3	1	13	3	17

Accidental poisonings and exposure to alcohol	0	0	4	2	4	3	9	3	13	2	10	0	40	10	50

Drowning	0	0	8	1	6	0	5	0	5	0	3	0	27	1	30

Other Unintentional injuries	0	0	27	3	27	1	27	1	42	3	31	3	154	11	166

Total	0	0	261	56	208	19	162	10	221	20	138	14	990	119	1115

Intentional injuries															

Self-inflicted injuries	0	0	80	16	89	7	107	8	124	13	58	5	458	49	507

Intentional self-poisoning by and exposure to alcohol	0	0	0	0	0	0	2	1	2	0	0	1	4	2	6

Homicide	0	0	24	3	25	2	12	1	9	1	6	1	76	8	83

Other intentional injuries	0	0	1	0	1	0	0	0	1	0	0	0	3	0	3

Total	0	0	105	19	115	9	121	10	136	14	64	7	541	59	599

Infectious and parasitic diseases															

Total (tuberculosis)	0	0	0	0	0	0	1	0	2	1	2	1	5	2	8

Respiratory infections															

Total (pneumonia)	0	0	1	1	1	1	6	2	12	5	22	12	42	21	63

Ethanol and methanol toxicity, undetermined intent	1	0	0	0	2	1	11	3	6	1	4	0	24	5	29

A large variation was observed for the average age of death by category and by sex. The average age of alcohol-attributable death was 47 years (46 years for men and 50 years for women). The youngest average age of death attributable to alcohol (excluding conditions arising during the perinatal period) resulted from motor vehicle accidents (35 years for men and 29 years for women). With respect to alcohol-prevented deaths, the mean age was 54 years (54 years for men and 53 years for women).

In Canada in 2005, a net total of 134,555 (95% CI: 36,690 to 236,376) potential years of life were lost for individuals between the ages of 0 and 64 years (see Table 4 for the number of PYLL attributable to alcohol by gender and age in Canada in 2005). This constituted 8.0% (95% CI: 2.2% to 14.1%) of the PYLLs in Canada for individuals between 0 and 64 years of age. Of these PYLLs, 11,536 (95% CI: 16 to 56,527) were prevented by alcohol, while 146,091 (95% CI: 93,217 to 236,392) were attributable to alcohol. The greatest specific contributor to alcohol-attributable PYLLs was motor vehicle accidents at 29,955 PYLLs (25,874 males, 4,081 females), while unintentional injuries totalled 46,861 PYLLs (40,812 males, 6,049 females). The greatest contributor to prevented PYLLs attributable to alcohol was ischemic heart disease with 9,774 PYLLs prevented (8,185 males, and 1,589 females).

**Table 4:** Potential years of life lost (PYLLs) attributable to alcohol in Canada in 2005 by gender and age

Gender	Age groups (years)	PYLLs	Lower 95% CI	Upper 95% CI
Women	0-14	313	98	528

	15-24	5244	30	14414
	
	25-34	2614	1144	4084
	
	35-44	4456	2656	6256
	
	45-54	11577	5735	17418
	
	55-64	10347	3495	17199
	
	Total	34551	13160	59899

Alcohol-attributable years of life lost per 100 000 women = 706				

Men	0-14	397	176	618

	15-24	21744	10639	32849
	
	25-34	17268	7779	26756
	
	35-44	16551	5848	27255
	
	45-54	24541	2635	46447
	
	55-64	19503	-3546	42552
	
	Total	100004	23531	176478

Alcohol-attributable years of life lost per 100 000 men = 249				

Total PYLLs	134555	36690	236376	

Overall, alcohol impacted men more than women; 9.3% (95% CI: 1.3%-17.2%) of all deaths and 10.0% (95% CI: 2.4%-17.7%) of all PYLLs among men were attributable to alcohol consumption. In contrast, 5.0% (95% CI: 1.9%-8.7%) of all deaths and 5.1% (95% CI: 2.0%-8.5%) of all PYLLs among women were attributable to alcohol consumption.

Table 5 presents for the years 1992, 2002 and 2005 in Canada 1) the gross number of deaths, 2) the number of deaths attributable to alcohol among all individuals and among individuals younger than 70 years of age, and 3) the PYLLs attributable to alcohol. A percentage increase in the gross number of deaths was not observed from 2002 to 2005; however, the number of deaths attributable to alcohol among individuals younger than 70 years of age, as well as the total number of PYLLs attributable to alcohol, increased between 1992 and 2005. Correspondingly, the *per capita *consumption of recorded and unrecorded alcohol increased from 9.20 l per person per year in 2002 to 9.32 l per person per year in 2005. 1992 data showed an even greater per capita consumption of recorded and unrecorded alcohol (9.58 l per person per year); however, it is important to note that unrecorded consumption was based on current estimates and not on population estimates for 1992.

**Table 5:** Mortality differences attributable to alcohol for different methodologies and times for Canada and other countries

Country	Study	Methods	Year	Alcohol- attributable deaths	% of all Deaths	All deaths caused under 70*	% of all deaths caused under 70	PYLLs*	% of all PYLLs
Canada*	Single et al., 1996 [10]	Single et al., 1996 [10]	1992	6,701	3.41%	4,913	6.64%	186,257	6.05%

Canada*	Patra et al., 2007 [11]	Rehm et al., 2006 [12]	2002	8,103	3.62%	5,061	7.56%	191,136	6.18%

Canada*	Current Study	Rehm et al., 2006 [12]	2005	8,312	3.61%	5,338	8.13%	197,040	6.25%

Canada*	Current Study	Current Study	2005	10,284	4.47%	5,613	8.55%	209,084	6.63%

Canada	Current Study	Rehm et al., 2006 [12]	2005	6,454	2.80%	-	-	171,700	5.45%

America A (WHO region AMR A)	Rehm et al., 2008 [Rehm et al., 2010]	Rehm et al., 2006 [12]	2004	55,000	1.99%	-	-	1,224,000	6.09%

Americas (WHO region AMR)	Rehm et al., 2008 [Rehm et al., 2010]	Rehm et al., 2006 [12]	2004	347,000	5.60%	-	-	6,845,000	10.21%

World	Rehm et al., 2010 [Rehm et al., 2010]	Rehm et al., 2006 [12]	2004	2,255,000	3.80%	-	-	40,863,000	4.38%

* Estimates are based on the gross number of deaths caused by alcohol and do not include those prevented except for: America A, Americas, and world

Table 5 also outlines the number of deaths and PYLLs attributable to alcohol consumption for the WHO regions of America A (Canada, Cuba and the United States) and the Americas, and for the world as a whole. In 2005, of the total deaths in Canada, a higher percentage of these deaths (all ages) were attributable to alcohol consumption than was the case for America A in 2004, but Canada had a lower percentage of total deaths in 2005 attributable to alcohol consumption than was observed for the Americas and for the world as a whole in 2004. In 2005 Canada had a lower percentage of the total PYLLs which were attributable to alcohol consumption than was the case for America A, the Americas, and the world as a whole in 2004.

## Discussion

Alcohol consumption has a significant negative impact on public health. Alcohol accounted for far more deaths than the number of deaths prevented by alcohol for Canadians

0 to 64 years of age. This paper improves upon previous estimates of mortality by using new methodology to 1) account for the undercoverage of alcohol consumption in the CADUMS 2008, which represented only 34% of recorded alcohol consumption in Canada, 2) take into consideration the right-skewed distribution of alcohol consumption, and 3) calculate 95% CIs. Additionally, we used updated risk ratio estimates in this study, as well as estimates for mortality for new disease categories such as colon cancer, rectal cancer, and tuberculosis [8,9]. Implementation of these new methods described by Rehm and colleagues [10] resulted in a higher estimated mortality attributable to alcohol compared to previous methods due primarily to the additional disease categories included in our analysis. The inclusion of new disease categories and use of new methodology allows for a more accurate characterization of alcohol-attributable mortality, which can potentially lead to better public health policies aimed at reducing alcohol-attributable harms.

This paper's analysis has its limitations. For our study we did not include all aspects of harms caused by the drinking of others, such as due to motor vehicle accidents and workplace injuries, which recently have been shown to constitute a large proportion of the burden of injury attributable to alcohol, as no meta-analyses exist that describe an RR which can be used to calculate this burden [25]. Due to the absence of available data on the incidence of certain events, such as the incidence of fetal loss during pregnancy attributable to alcohol consumption, our analysis was restricted to deaths and PYLLs and does not provide a complete picture of the effects of alcohol on all aspects of health. Additionally, we did not estimate the number of deaths for Canadians over 65 years of age since specified causes of death for people in this age group were considered to be unreliable. If we had included in our analysis the number of deaths for Canadians aged 65 years and older, the number of net deaths attributable to alcohol would have risen from 3,969 to 8,953 and the number of PYLLs would have risen from 134,555 to 172,255; however, as indicated above, primary information concerning the causes of death for Canadians over 65 years of age is considered to be unreliable.

For this study, we used cross-sectional estimates of the average volume of alcohol consumption, not taking into consideration individuals' past and current patterns of drinking as distinct from volume, with the exception of injury which incorporated binge drinking estimates. While restrictions on volume may not be important for certain diseases, patterns of drinking impact the risk of diseases such as ischemic heart disease and other cardiovascular diseases [4,26,27]. In addition, not including past patterns of drinking is problematic for studying the role of alcohol in a person's developing malignant neoplasms as the time between exposure to a carcinogen and exhibiting symptoms of cancer can range from between 15 and 30 years [28]. Liver cirrhosis at the individual level is mainly affected by long-term heavy drinking; however, immediate effects decreasing its incidence are seen at the country level after a short time when alcohol consumption decreases dramatically [29]. Accordingly, public policies aimed at reducing alcohol-attributable harms will not only have an immediate impact on alcohol-related harms but will also have long-term benefits.

Limitations in the study design, sampling, and response rates of the CADUMS 2008 led to an undercoverage of the resulting estimates of alcohol consumption in Canada. For example, the CADUMS 2008 excluded people living in the Northwest Territories (which have a greater proportion of individuals suffering from alcohol abuse problems compared to the rest of Canada), and excluded homeless and institutionalized individuals, as well as those individuals without a home phone. The inherent problems with the CADUMS 2008 were adjusted for in our study by triangulating the survey data with the *per capita *alcohol consumption estimate (according to the methods of Rehm and colleagues [10]).

The response rate for the CADUMS 2008 survey was relatively low, with an overall response rate of 36.5%. However, it has been previously demonstrated that higher response rates do not necessarily change the resulting distribution of alcohol consumption when the response rate varies between 30% - 60% and similar sampling methods are used [19,30]. Moreover, since we triangulated survey data with *per capita *consumption data for our study, underreporting of alcohol consumption should not be problematic [31].

Undercoverage of alcohol may be present in both the survey alcohol exposure data and the studies used in the meta-analyses to calculate the RR functions. This undercoverage may lead to an overestimation of the alcohol RR functions for all diseases; however, the effects of these two instances of undercoverage differ. Only undercoverage from measurement error (response bias and construct validity) affects the RR functions. Undercoverage in exposure estimates derived from survey data arises from measurement error, non-response to the alcohol survey, and from the exclusion of certain populations from the survey. Since we do not know the extent of undercoverage caused by measurement error in the studies that were used in the meta-analyses, it is impossible to determine the coverage rate to which our survey data should be standardized in order to correct the RR functions. Since it is very likely that the undercoverage in epidemiological studies is much less than in population surveys [32], we corrected the undercoverage of the CADUMS to 90% of the *per capita *consumption in order to account for the overestimated RRs calculated from observational studies.

If we did not adjust for the undercoverage of the survey data, the number of deaths attributable to alcohol consumption would be 47.9% of the total number of deaths calculated after adjustment to 90% of *per capita *consumption, resulting in a calculation of 1,896 net deaths from alcohol. It should be noted, however, that adjustment of *per capita *consumption data across countries is necessary if the mortality figures are to be comparable across countries.

Our study is also limited by the RRs used to calculate the number of alcohol- attributable deaths and PYLLs in Canada for 2004. These limitations stem from the meta-analyses of the adjusted RRs [33]. There are two potential problems with the RR functions that could affect the results of our study. First, there is an assumption that RRs for alcohol consumption are stable across countries. While this assumption is plausible for chronic diseases, which are mainly determined by biological relationships (as demonstrated by the low heterogeneity in the chronic disease RR meta-analyses), this assumption is less plausible for injuries where social influences will affect the RRs (e.g. traffic injury depends on highway safety standards, density of cars, etc.). As most of the RRs for injury are estimated based on data from high-income countries with similar environmental conditions to Canada, these RR estimates are valid for our study. Second, the use of adjusted RRs may overestimate the true RRs as observed by Flegal and others [34-36]; however, the size of the potential bias has recently been shown to be relatively small [37].

Regardless of our study's limitations and assumptions, alcohol is clearly a major contributor to mortality and PYLLs, and has a significant public health impact in Canada. In contrast to tobacco-related mortality, the average age of death resulting from alcohol consumption is much lower [2,13], such that mortality due to alcohol consumption occurs primarily in adults of working age and results in a negative economic impact. This was also observed in our study, as alcohol was responsible for 7.7% of all deaths and 8.0% of all PYLLs for people between 0 and 64 years of age.

A monitoring system on alcohol consumption and alcohol-related harms is imperative as the burden of alcohol-attributable harms in Canada is unnecessary and could be reduced in a relatively short period of time if effective public health policies were implemented [38,39]. These policies could include taxation, additional and/or stronger sanctions to combat impaired driving, and specific measures to decrease aggression and violence [39-41]. Rehm and colleagues analyzed the effects of these interventions in Canada for 2002 and found that an increase in price of 25% would result in a decrease of 59 net deaths and more than 1,500 net PYLLs [42,43]. By reducing in Canada the maximum acceptable blood alcohol content while operating a motor vehicle from 0.08% to 0.05%, Rehm and colleagues found that there would be 173 fewer net deaths and more than 7,000 fewer net PYLLs (a 4.1% reduction and a 5.0% reduction, respectively from the baseline scenario) [42,43]. A real world example of government policy affecting mortality due to alcohol consumption was observed in Russia during the Gorbachev anti-alcohol campaign, where annual *per capita *consumption of pure alcohol fell from 14.2 l in 1984 to 10.7 l in 1987. As a result of this decrease in alcohol consumption, all cause mortality rates for 40-44 year olds decreased by 39% for men and by 29% for women and alcohol-related mortality rates for 40-44 year olds decreased by 63% for men and by 63% for women between 1984 and 1987 [44]. Each of the policy measures suggested above has been shown to be effective in jurisdictions outside of Canada which face similar epidemiological profiles of mortality due to alcohol consumption [39-41,45]. For the above Canadian examples, the noted reductions in mortality represent a net estimate and, thus, incorporate potential increases in the incidence of diseases where alcohol exhibits a protective effect.

A comprehensive alcohol consumption and alcohol-attributable harms monitoring system in Canada would allow for evaluation of the effectiveness of public health policies and, once established, could be used to identify those areas where targeted alcohol policies would have the greatest impact. Finally, such a system would help to estimate alcohol-attributable harms in the future using time-trend analysis. Monitoring systems on the harms of alcohol should include the resulting social harms. In some regions it is possible that the social harms associated with alcohol consumption may exceed the burden of mortality.

## Conclusion

The estimated mortality and PYLLs attributable to alcohol consumption in Canada in 2005 is substantial, with alcohol-attributable deaths from motor vehicle accidents, malignant neoplasm and digestive diseases being the largest contributors to this burden. The novel methods described in this paper allow for the most accurate characterization of the burden of mortality attributable to alcohol, and are directly comparable to estimates from other countries that use the same method of adjusting alcohol consumption and to estimates obtained in GBD reports. As outlined in the World Health Assembly strategy to reduce the harmful use of alcohol, it is imperative to quantify and monitor the burden of mortality due to alcohol consumption in order to formulate and evaluate public health policies aimed at reducing alcohol's harmful effects. Accordingly, we propose that the estimates presented in this paper act as a baseline measurement for a monitoring system for Canada.

## Competing interests

The authors declare that they have no competing interests.

## Authors' contributions

Kevin Shield and Jürgen Rehm participated in the design and conceptualization of the article. Kevin Shield, Benjamin Taylor, Jürgen Rehm and Tara Kehoe conceptualized the statistical methodology for the project. Kevin Shield performed the statistical analysis and drafted the manuscript. All authors have revised the manuscript critically for important intellectual content and have approved the final manuscript.

## Pre-publication history

The pre-publication history for this paper can be accessed here:

http://www.biomedcentral.com/1471-2458/12/91/prepub

## Supplementary Material

Additional file 1 **Outlines the sources for the RRs by ICD 10 code **[46-62]. Click here for file

## References

[B1] World Health Organization Global Health Risks. Mortality and burden of disease attributable to selected major risks 2009 Geneva, Switzerland: World Health Organization

[B2] Rehm J Mathers C Popova S Thavorncharoensap M Teerawattananon Y Patra J Global burden of disease and injury and economic cost attributable to alcohol use and alcohol-use disorders Lancet 2009 373 2223 2233 10.1016/S0140-6736(09)60746-7 19560604

[B3] Rehm J Baliunas D Borges GLG Graham K Irving HM Kehoe T Parry CD Patra J Popova L Poznyak V Roerecke M Room R Samokhvalov AV Taylor B The relation between different dimensions of alcohol consumption and burden of disease - an overview Addiction 2010 105 817 843 10.1111/j.1360-0443.2010.02899.x 20331573 PMC3306013

[B4] Rehm J Room R Graham K Monteiro M Gmel G Sempos C The relationship of average volume of alcohol consumption and patterns of drinking to burden of disease - an overview Addiction 2003 98 1209 1228 10.1046/j.1360-0443.2003.00467.x 12930209

[B5] English DR Holman CDJ Milne E Winter MJ Hulse GK Codde G Bower CI Cortu B de Klerk N Lewin GF Knuiman M Kurinczuk JJ Ryan GA The quantification of drug caused morbidity and mortality in Australia 1995 1995 Canberra, Australia: Australian Government Publishing Service

[B6] World Health Organization World Health Organization: Global strategy to reduce the harmful use of alcohol 2010 Geneva, Switzerland: World Health Organization 10.2471/BLT.19.241737 PMC7047030 32132758

[B7] Rehm J Room R Monitoring of alcohol use and attributable harm from an international perspective Contemp Drug Probl 2009 36 575 588

[B8] Rehm J Patra J Popova S Alcohol-attributable mortality and potential years of life lost in Canada 2001: implications for prevention and policy Addiction 2006 101 373 384 10.1111/j.1360-0443.2005.01338.x 16499510

[B9] Rehm J Taylor B Patra J Volume of alcohol consumption, patterns of drinking and burden of disease in the European region 2002 Addiction 2006 101 1086 1095 10.1111/j.1360-0443.2006.01491.x 16869838

[B10] Rehm J Kehoe T Gmel G Stinson F Grant B Gmel G Statistical modelling of volume of alcohol exposure for epidemiological studies of population health: the example of the US Popul Health Metr 2010 8 3 10.1186/1478-7954-8-3 20202213 PMC2841092

[B11] Gmel G Kuntsche E Wicki M Labhart F Measuring Alcohol-Related Consequences in School Surveys: Alcohol-Attributable Consequences or Consequences with students' alcohol attribution Am J Epidemiol 2010 171 94 104 10.1093/aje/kwp331 19969527

[B12] Murray CJL Lopez AD Black R Mathers CD Shibuya K Ezzati M Salomon JA Michaud CM Walker N Vos T Global burden of disease 2005: call for collaborators Lancet 2007 370 109 110 10.1016/S0140-6736(07)61064-2 17630021

[B13] Single E Robson L Rehm J Xie X Morbidity and mortality attributable to alcohol, tobacco, and illicit drug use in Canada Am J Public Health 1999 89 385 390 10.2105/AJPH.89.3.385 10076491 PMC1508614

[B14] Health Canada Canadian Alcohol and Drug Use Monitoring Survey http://www.hc-sc.gc.ca/hc-ps/drugs-drogues/cadums-esccad-eng.php

[B15] Health Canada Canadian Alcohol and Drug Use Monitoring Survey 2008: Microdata User Guide http://prod.library.utoronto.ca/datalib/codebooks/cstdli/cadums/2008/cadums-technical-guide-2008-final-eng.pdf

[B16] Canadian Centre on Substance Abuse Canadian Addiction Survey 2004: Microdata eGuide 2004 Ottawa, Canada: Canadian Centre on Substance Abuse

[B17] National Institute on Alcohol Abuse and Alcoholism National Epidemiologic Survey on Alcohol and Related Conditions Alcohol 2006 National Institute on Alcohol Abuse and Alcoholism http://pubs.niaaa.nih.gov/publications/AA70/AA70.htm

[B18] Public Health Agency of Canada Alcohol use and pregnancy: an important Canadian public health and social issue 2007 Ottawa, Canada: Public Health Agency of Canada

[B19] Gmel G Rehm J Measuring alcohol consumption Contemp Drug Probl 2004 31 467 540

[B20] Macdonald S Wells S Giesbrecht N Unrecorded alcohol consumption in Ontario, Canada: estimation procedures and research implications Drug Alcohol Rev 1999 18 21 29 10.1080/09595239996725

[B21] Statistics Canada Vital Statistics-Death Database 2011 http://www.statcan.gc.ca/cgi-bin/imdb/p2SV.pl?Function=getSurvey&SDDS = 3233&lang=en&db=imdb&adm=8&dis=2

[B22] Mathers C Stein C Ma Fat D Rao C Inoue M Tomijima N Bernard B Lopez AD Murray CJL Global Burden of Disease 2000: version 2 methods and results 2002 Geneva, Switzerland: World Health Organization

[B23] Taylor B Rehm J Room R Patra J Bondy S Determination of lifetime injury mortality risk in Canada in 2002 by drinking amount per occasion and number of occasions Am J Epidemiol 2008 168 1119 1125 10.1093/aje/kwn215 18718895

[B24] Rehm J Gmel Gj Shield K Frick U Kehoe T Gmel Gs Estimating uncertainty for alcohol-attributable fractions for infectious and chronic disease 2010 Toronto, Canada: Centre for Addiction and Mental Health 10.1186/1471-2288-11-48 PMC3088897 21496313

[B25] Laslett AM Catalano P Chikritzhs T Dale C Doran C Ferris J Jainullabudeen T Livingston M Matthews S Mugavin S Room, Schlotterleinand M, Wilkinson C: The range and magnitude of alcohol's harm to others 2010 Fitzroy, Australia: Turning Point Alcohol & Drug Centre

[B26] Roerecke M Rehm J Irregular heavy drinking occasions and risk of ischemic heart disease: a systematic review and meta-analysis Am J Epidemiol 2010 171 633 644 10.1093/aje/kwp451 20142394

[B27] Murray R Connett J Tyas S Bond R Ekuma O Silversides C Barnes G Alcohol volume, drinking pattern, and cardiovascular disease morbidity and mortality: is there a U-shaped function? Am J Epidemiol 2002 155 242 248 10.1093/aje/155.3.242 11821249

[B28] Rehm J Patra J Popova L Alcohol drinking cessation and its effect on oesophageal and head and neck cancers: a pooled analysis Int J Cancer 2007 121 1132 1137 10.1002/ijc.22798 17487833

[B29] Zatonski W Sulkowska U Manczuk M Rehm J Lowenfels AB La Vecchia C Liver cirrhosis mortality in Europe, with special attention to central and eastern Europe Eur Addict Res 2010 16 193 201 10.1159/000317248 20606444

[B30] Kehoe T Gmel G Shield K Gmel G Rehm J Determining the best population-level alcohol consumption model and its impact on estimates of alcohol-attributable harms Popul Health Metr in press 10.1186/1478-7954-10-6 PMC3352241 22490226

[B31] Rehm J Klotsche J Patra J Comparative quantification of alcohol exposure as risk factor for global burden of disease Int J Methods Psychiatr Res 2007 16 66 76 10.1002/mpr.204 17623386 PMC6878477

[B32] Shield K Rehm J Difficulties with telephone-based surveys on alcohol in high-income countries: the Canadian example Int J Methods Psychiatr Res in press 10.1002/mpr.1345 PMC3561771 22337654

[B33] Rehm J Baliunas D Borges GLG Graham K Irving HM Kehoe T Parry CD Patra J Popova L Poznyak V Roerecke M Room R Samokhvalov AV Taylor B The relation between different dimensions of alcohol consumption and burden of disease - An overview Addiction 2010 105 817 843 10.1111/j.1360-0443.2010.02899.x 20331573 PMC3306013

[B34] Flegal KM Williamson DF Graubard BI Using adjusted relative risks to calculate attributable fractions Am J Public Health 2006 96 398 10.2105/AJPH.2005.079731 16449574 PMC1470504

[B35] Korn EL Graubard BI Analysis of Health Surveys 1999 New York, United States: John Wiley & Sons Inc

[B36] Rockhill B Newman B Use and misuse of population attributable fractions Am J Public Health 1998 88 15 19 10.2105/AJPH.88.1.15 9584027 PMC1508384

[B37] Baliunas D A comparison of two methods of adjusted attributable fraction estimation as applied to the four major smoking related causes of death in Canada, in 2005 PhD Thesis 2011 University of Toronto, Dalla Lana School of Public Health

[B38] World Health Organization The world health report 2002: Reducing risks, promoting healthy life 2002 Geneva, Switzerland: World Health Organization

[B39] Chisholm D Rehm J van Ommeren M Monteiro M Reducing the global burden of hazardous alcohol use: a comparative cost-effectiveness analysis J Stud Alcohol 2004 65 782 793 15700517 10.15288/jsa.2004.65.782

[B40] Babor T Caetano R Casswell S Edwards G Giesbrecht N Graham K Grube J Gruenewald P Hill L Holder H Livingston M Österberg E Rehm J Room R Rossow I Alcohol: No ordinary commodity. Research and public policy 2010 2 Oxford and London, United Kingdom of Great Britain and Northern Ireland: Oxford University Press

[B41] Room R Babor T Rehm J Alcohol and public health: a review Lancet 2005 365 519 530 15705462 10.1016/S0140-6736(05)17870-2

[B42] Rehm J Gnam W Popova S Patra J Sarnocinska-Hart A Avoidable cost of alcohol abuse in Canada 2002 2008 Toronto, Canada: Centre for Addiction and Mental Health 10.1159/000321463 21150206

[B43] Rehm J Patra J Gnam W Sarnocinska-Hart A Popova S Avoidable cost of alcohol abuse in Canada Eur Addict Res 2011 17 72 79 10.1159/000321463 21150206

[B44] Leon D Chenet L Shkolnikov V Zakharov S Shapiro J Rakhmanova G Vassin S McKee M Huge variation in Russian mortality rates 1984-1994: Artefact, alcohol, or what? Lancet 1997 350 383 388 10.1016/S0140-6736(97)03360-6 9259651

[B45] Wagenaar AC Maldonado-Molina MM Wagenaar BA Effects of alcohol tax increases on alcohol-related disease mortality in Alaska: time-series analyses from 1976 to 2004 Am J Public Health 2009 99 1464 1470 10.2105/AJPH.2007.131326 19008507 PMC2707462

[B46] Lönnroth K Williams B Stadlin S Jaramillo E Dye C Alcohol use as a risk factor for tuberculosis - a systematic review BMC Public Health 2008 8 289 10.1186/1471-2458-8-289 18702821 PMC2533327

[B47] Rehm J Samokhvalov AV Neuman MG Room R Parry CD Lönnroth K Patra J Poznyak V Popova S The association between alcohol use, alcohol use disorders and tuberculosis (TB) A systematic review BMC Public Health 2009 9 450 10.1186/1471-2458-9-450 PMC2796667 19961618

[B48] Baan R Straif K Grosse Y Secretan B El Ghissassi F Bouvard V Alteri A Cogliano V on behalf of the WHO international agency for research on cancer monograph working group Carcinogenicity of alcoholic beverages Lancet Oncol 2007 8 292 293 10.1016/S1470-2045(07)70099-2 17431955

[B49] Corrao G Bagnardi V Zambon A La Vecchia C A meta-analysis of alcohol consumption and the risk of 15 diseases Prev Med 2004 38 613 619 10.1016/j.ypmed.2003.11.027 15066364

[B50] Baliunas D Taylor B Irving H Roerecke M Patra J Mohapatra S Rehm J Alcohol as a risk factor for type 2 diabetes - a systematic review and meta-analysis Diabetes Care 2009 32 2123 2132 10.2337/dc09-0227 19875607 PMC2768203

[B51] Samokhvalov AV Irving H Mohapatra S Rehm J Alcohol consumption, unprovoked seizures and epilepsy: a systematic review and meta-analysis Epilepsia 2010 51 1177 1184 10.1111/j.1528-1167.2009.02426.x 20074233

[B52] Taylor B Irving HM Baliunas D Roerecke M Patra J Mohapatra S Rehm J Alcohol and hypertension: gender differences in dose-response relationships determined through systematic review and meta-analysis Addiction 2009 104 1981 1990 10.1111/j.1360-0443.2009.02694.x 19804464

[B53] Roerecke M Rehm J Alcohol consumption and the risk for morbidity and mortality of ischemic heart disease - A systemic review and meta-analysis 2010 Toronto, Canada: Centre for Addiction and Mental Health

[B54] Samokhvalov AV Irving HM Rehm J Alcohol as a risk factor for atrial fibrillation: a systematic review and meta-analysis Eur J Cardiovasc Prev Rehabil 2010 17 706 712 10.1097/HJR.0b013e32833a1947 21461366 PMC3065072

[B55] Patra J Taylor B Irving H Roerecke M Baliunas D Mohapatra S Rehm J Alcohol consumption and the risk of morbidity and mortality for different stroke types - a systematic review and meta-analysis BMC Public Health 2010 10 258 10.1186/1471-2458-10-258 20482788 PMC2888740

[B56] Rehm J Taylor B Mohapatra S Irving H Baliunas D Patra J Roerecke M Alcohol as a risk factor for liver cirrhosis - a systematic review and meta-analysis Drug Alcohol Rev 2010 29 437 445 10.1111/j.1465-3362.2009.00153.x 20636661

[B57] Irving HM Samokhvalov A Rehm J Alcohol as a risk factor for pancreatitis A systematic review and meta-analysis JOP 2009 10 387 392 PMC3299482 19581740

[B58] Samokhvalov AV Irving HM Rehm J Alcohol consumption as a risk factor for pneumonia: systematic review and meta-analysis Epidemiol Infect 2010 138 1789 1795 10.1017/S0950268810000774 20380771

[B59] Patra J Bakker R Irving H Jaddoe VWV Rehm J Dose-response relationship between alcohol consumption before and during pregnancy and the risks of low birthweight, preterm birth and small for gestational age (SGA)-a systematic review and meta-analyses BJOG 2011 188 1411 1421 10.1111/j.1471-0528.2011.03050.x PMC3394156 21729235

[B60] Taylor B Irving HM Kanteres F Room R Borges G Cherpitel C Greenfield T Rehm J The more you drink, the harder you fall: a systematic review and meta-analysis of how acute alcohol consumption and injury or collision risk increase together Drug Alcohol Depend 2010 110 108 116 10.1016/j.drugalcdep.2010.02.011 20236774 PMC2887748

[B61] Rehm J Room R Taylor B Method for moderation: measuring lifetime risk of alcohol-attributable mortality as a basis for drinking guidelines Int J Methods Psychiatr Res 2008 17 141 151 10.1002/mpr.259 18763694 PMC6878565

[B62] Taylor B Rehm J Room R Patra J Bondy S Determination of lifetime injury mortality risk in Canada in 2002 by drinking amount per occasion and number of occasions Am J Epidemiol 2008 168 1119 1125 10.1093/aje/kwn215 18718895

