# Supplementary material for: Mortality and potential years of life lost attributable to alcohol consumption in Canada in 2005
Source: BMC Public Health. 2012 Jan 31;12:91. doi: 10.1186/1471-2458-12-91 (PMC3305515; doi:10.1186/1471-2458-12-91)
Supplement: Additional file 1 — Outlines the sources for the RRs by ICD 10 code [46-62]. [file 1471-2458-12-91-S1.DOCX]

Appendix

| Table 1. Categories of alcohol-related disease and sources used for determining alcohol-attributable fractions | | | |
| --- | --- | --- | --- |
|  |  |  |  |
| Condition | | ICD 10 Code | Source for AAF |
| Infectious and parasitic diseases | |  |  |
|  | Tuberculosis | A15-A19 | Lönnroth et al., 2008 [1] (causal relationship see: Rehm et al., 2009[2]) |
| Malignant neoplasm's | |  |  |
|  | Mouth and oropharynx cancers | C00-C14 | Baan R et al., 2004 [3] (based on Relative risks from Corrao et al., 2004) [4] |
|  | Esophageal cancer | C15 | Baan R et al., 2004 [3] (based on Relative risks from Corrao et al., 2004) [4] |
|  | Liver cancer | C22 | Baan R et al., 2004 [3] (based on Relative risks from Corrao et al., 2004) [4] |
|  | Laryngeal cancer | C32 | Baan R et al., 2004 [3] (based on Relative risks from Corrao et al., 2004) [4] |
|  | Breast cancer | C50 | Baan R et al., 2004 [3] (based on Relative risks from Corrao et al., 2004) [4] |
|  | Colon cancer | C18 | Baan R et al., 2004 [3] (based on Relative risks from Corrao et al., 2004) [4] |
|  | Rectal cancer | C20 | Baan R et al., 2004 [3] (based on Relative risks from Corrao et al., 2004) [4] |
| Diabetes | |  |  |
|  | Diabetes mellitus | E10-E14 | Baliunas et al., 2009 [5] |
| Neuro-psychiatric conditions | |  |  |
|  | Alcoholic psychoses | F10.0, F10.3-F10.9 | 100% AAF per definition |
|  | Alcohol abuse | F10.1 | 100% AAF per definition |
|  | Alcohol dependence syndrome | F10.2 | 100% AAF per definition |
|  | Degeneration of nervous system due to alcohol | G31.2 | 100% AAF per definition |
|  | Epilepsy | G40-G41 | Samokhvalov et al., 2010 [6] |
|  | Alcohol polyneuropathy | G62.1 | 100% AAF per definition |
| Cardiovascular disease | |  |  |
|  | Hypertensive disease | I10-I15 | Taylor et al., 2010[7] |
|  | Ischemic heart disease | I20-I25 | Roerecke et al., 2011 [8] |
|  | Alcohol cardiomyopathy | I42.6 | 100% AAF per definition |
|  | Cardiac arrhythmias | I47-I49 | Samokhvalov et al., 2010 [9] |
|  | Ischemic stroke | I60-I62 | Patra et al., 2010 [10] |
|  | Hemorrhagic and other non-ischemic stroke | I63-I66 | Patra et al., 2010 [10] |
| Digestive diseases | |  |  |
|  | Alcoholic gastritis | K29.2 | 100% AAF per definition |
|  | Cirrhosis of the liver | K70, K74 | Rehm et al., 2010 [11] |
|  | Acute and chronic pancreatitis | K85, K86.1 | Irving et al., 2009 [12] |
|  | Chronic pancreatitis (alcohol-induced) | K86.0 | 100% AAF per definition |
| Respiratory infections | |  |  |
|  | Pneumonia | J10.0, J11.0, J12-J15, J18 | Samokhvalov et al., 2010 [13] |
| Conditions arising during the prenatal period | |  |  |
|  | Low birth weight: as defined by the global burden of disease | P05-P07 | Patra et al., 2011 [14] |
|  | Fetal alcohol syndrome (dysmorphic) | Q86.0 | 100% AAF per definition |
|  | Excess alcohol blood levels | R78.0 | 100% AAF per definition |
| Unintentional injuries | |  |  |
|  | Motor vehicle accidents | § | Taylor et al., 2010 [15] for relative risk, Rehm et al., 2008 [16] and Taylor et al., 2008 [17] for AAF calculation methods |
|  | Poisonings | X40-X49 | Taylor et al., 2010 [15] for relative risk, Rehm et al., 2008 [16] and Taylor et al., 2008 [17] for AAF calculation methods |
|  | Falls | W00-W19 | Taylor et al., 2010 [15] for relative risk, Rehm et al., 2008 [16] and Taylor et al., 2008 [17] for AAF calculation methods |
|  | Fires | X00-X09 | Taylor et al., 2010 [15] for relative risk, Rehm et al., 2008 [16] and Taylor et al., 2008 [17] for AAF calculation methods |
|  | Accidental Poisonings and exposure to alcohol | X45 | 100% AAF per definition |
|  | Drowning | W65-W74 | Taylor et al., 2010 [15] for relative risk, Rehm et al., 2008 [16] and Taylor et al., 2008 [17] for AAF calculation methods |
|  | Other Unintentional injuries | †Rest of V-series and W20-W64, W 75-W99, X10-X39, X50-X59, Y40-Y86, Y88, and Y89 | Taylor et al., 2010 [15] for relative risk, Rehm et al., 2008 [16] and Taylor et al., 2008 [17] for AAF calculation methods |
| Intentional injuries | |  | Taylor et al., 2010 [15] for relative risk, Rehm et al., 2008 [16] and Taylor et al., 2008 [17] for AAF calculation methods |
|  | Self-inflicted injuries | X60-X84 and Y87.0 | Taylor et al., 2010 [15] for relative risk, Rehm et al., 2008 [16] and Taylor et al., 2008 [17] for AAF calculation methods |
|  | Intentional self-poisoning by and exposure to alcohol | X65 | 100% AAF per definition |
|  | Homicide | X85-Y09, Y87.1 | Taylor et al., 2010 [15] for relative risk, Rehm et al., 2008 [16] and Taylor et al., 2008 [17] for AAF calculation methods |
|  | Other intentional injuries |  | Taylor et al., 2010 [15] for relative risk, Rehm et al., 2008 [16] and Taylor et al., 2008 [17] for AAF calculation methods |
| Ethanol and methanol toxicity, undetermined intent | | Y15 | 100% AAF per definition |
| § V021–V029, V031–V039, V041–V049, V092, V093, V123–V129, V133–V139, V143–V149, V194–V196, V203–V209, V213–V219, V223–V229, V233–V239, V243–V249,V253–V259, V263–V269, V273– V279, V283–V289, V294–V299, V304–V309, V314–V319, V324–V329, V334–V339, V344–V349, V354–V359, V364–V369, V374–V379, V384–V389, V394–V399, V404–V409, V414–V419, V424–V429, V434–V439, V444–V449, V454–V459, V464– V469, V474–V479, V484–V489, V494–V499, V504–V509, V514–V519, V524–V529, V534–V539, V544–V549, V554–V559, V564–V569, V574–V579, V584–V589, V594–V599, V604–V609, V614–V619, V624–V629, V634–V639, V644–V649, V654– V659, V664–V669, V674–V679, V684–V689, V694–V699, V704–V709, V714–V719, V724–V729, V734–V739, V744–V749, V754–V759, V764–V769, V774–V779, V784–V789, V794–V799, V803–V805, V811, V821, V830–V833, V840–V843, V850– V853, V860–V863, V870–V878, V892. †Rest of V = V-series MINUS §. | | | |

Reference List

1. Lönnroth K, Williams B, Stadlin S, Jaramillo E, Dye C: **Alcohol use as a risk factor for tuberculosis - a systematic review.** *BMC Public Health* 2008, **8:**289.

2. Rehm J, Samokhvalov AV, Neuman MG, Room R, Parry CD, Lönnroth K, Patra J, Poznyak V, Popova S: **The association between alcohol use, alcohol use disorders and tuberculosis (TB). A systematic review.** *BMC Public Health* 2009, **9:**450.

3. Baan R, Straif K, Grosse Y, Secretan B, El Ghissassi F, Bouvard V, Alteri A, Cogliano V, on behalf of the WHO international agency for research on cancer monograph working group: **Carcinogenicity of alcoholic beverages.** *Lancet Oncol* 2007, **8:**292-293.

4. Corrao G, Bagnardi V, Zambon A, La Vecchia C: **A meta-analysis of alcohol consumption and the risk of 15 diseases.** *Prev Med* 2004, **38:**613-619.

5. Baliunas D, Taylor B, Irving H, Roerecke M, Patra J, Mohapatra S, Rehm J: **Alcohol as a risk factor for type 2 diabetes - a systematic review and meta-analysis.** *Diabetes Care* 2009, **32:**2123-2132.

6. Samokhvalov AV, Irving H, Mohapatra S, Rehm J: **Alcohol consumption, unprovoked seizures and epilepsy: a systematic review and meta-analysis.** *Epilepsia* 2010, **51:**1177-1184.

7. Taylor B, Irving HM, Baliunas D, Roerecke M, Patra J, Mohapatra S, Rehm J: **Alcohol and hypertension: gender differences in dose-response relationships determined through systematic review and meta-analysis.** *Addiction* 2009, **104:**1981-1990.

8. Roerecke M, Rehm J: *Alcohol consumption and the risk for morbidity and mortality of ischemic heart disease - A systemic review and meta-analysis.* Toronto, Canada: Centre for Addiction and Mental Health; 2010.

9. Samokhvalov AV, Irving HM, Rehm J: **Alcohol as a risk factor for atrial fibrillation: a systematic review and meta-analysis.** *Eur J Cardiovasc Prev Rehabil* 2010, **17:**706-712.

10. Patra J, Taylor B, Irving H, Roerecke M, Baliunas D, Mohapatra S, Rehm J: **Alcohol consumption and the risk of morbidity and mortality for different stroke types - a systematic review and meta-analysis.** *BMC Public Health* 2010, **10:**258.

11. Rehm J, Taylor B, Mohapatra S, Irving H, Baliunas D, Patra J, Roerecke M: **Alcohol as a risk factor for liver cirrhosis - a systematic review and meta-analysis.** *Drug Alcohol Rev* 2010, **29:**437-445.

12. Irving HM, Samokhvalov A, Rehm J: **Alcohol as a risk factor for pancreatitis. A systematic review and meta-analysis.** *JOP* 2009, **10:**387-392.

13. Samokhvalov AV, Irving HM, Rehm J: **Alcohol consumption as a risk factor for pneumonia: systematic review and meta-analysis.** *Epidemiol Infect.* 2010, **138**:1789-1795.

14. Patra J, Bakker R, Irving H, Jaddoe VWV, Rehm J. **Dose-response relationship between alcohol consumption before and during pregnancy and the risks of low birthweight, preterm birth and small for gestational age (SGA)-a systematic review and meta-analyses.** *BJOG,* 2011, **188**:1411-1421.

15. Taylor B, Irving HM, Kanteres F, Room R, Borges G, Cherpitel C, Greenfield T, Rehm J: **The more you drink, the harder you fall: a systematic review and meta-analysis of how acute alcohol consumption and injury or collision risk increase together.** *Drug Alcohol Depend* 2010, **110:**108-116.

16. Rehm J, Room R, Taylor B: **Method for moderation: measuring lifetime risk of alcohol-attributable mortality as a basis for drinking guidelines.** *Int J Methods Psychiatr Res* 2008, **17:**141-151.

17. Taylor B, Rehm J, Room R, Patra J, Bondy S: **Determination of lifetime injury mortality risk in Canada in 2002 by drinking amount per occasion and number of occasions.** *Am J Epidemiol* 2008, **168:**1119-1125.
